# Prevalence of MRI-detected mediopatellar plica in subjects with knee pain and the association with MRI-detected patellofemoral cartilage damage and bone marrow lesions: data from the Joints On Glucosamine study

**DOI:** 10.1186/1471-2474-14-292

**Published:** 2013-10-12

**Authors:** Daichi Hayashi, Li Xu, Ali Guermazi, C Kent Kwoh, Michael J Hannon, Mohamed Jarraya, Stephanie M Green, John M Jakicic, Carolyn E Moore, Frank W Roemer

**Affiliations:** 1 Quantitative Imaging Center, Department of Radiology, Boston University School of Medicine, FGH Building 3rd Floor, 820 Harrison Avenue, Boston, MA 02118, USA; 2 Department of Radiology, Bridgeport Hospital, Yale University School of Medicine, Bridgeport, CT 06610, USA; 3 Department of Radiology, Beijing Jishuitan Hospital, Beijing 100035, China; 4 Division of Rheumatology and Clinical Immunology, University of Pittsburgh School of Medicine, Pittsburgh, PA 15261, USA; 5 Pittsburgh VA Healthcare System, Pittsburgh, PA 15240, USA; 6 Department of Health and Physical Activity, University of Pittsburgh, Pittsburgh, PA 15260, USA; 7 Department of Nutrition and Food Science, Texas Woman’s University, Houston, TX 77030, USA; 8 Department of Radiology, University of Erlangen, Erlangen, Germany

**Keywords:** Mediopatellar plica, Cartilage, Bone marrow lesion, Knee, MRI

## Abstract

**Background:**

The mediopatellar plica is a synovial fold representing an embryonic remnant from the developmental process of the synovial cavity formation in the knee. We aimed to examine the frequency of MRI-detected mediopatellar plica and its cross-sectional association with MRI-detected cartilage damage and bone marrow lesions (BMLs) in the patellofemoral joint (PFJ) in a cohort of subjects with knee pain.

**Methods:**

342 knees with chronic frequent knee pain were evaluated for MRI-detected mediopatellar plica (type A, B or C according to the modified Sakakibara classification). Cartilage damage (scored 0 to 6) and BMLs (scored 0 to 3) were semiquantitatively assessed in four subregions of the PFJ on MRI. Hoffa-synovitis and effusion-synovitis were graded 0 to 3. Patellar length ratio (PLR), lateral patellar tilt angle (LPTA), bisect offset (BO), and sulcus angle (SA) were measured on MRI. The presence of mediopatellar plica and its association with cartilage damage and BMLs in the PFJ was assessed using logistic regression after adjusting for age, gender, body mass index, PLR, LPTA, BO, SA, and Hoffa- and effusion-synovitis.

**Results:**

163 (47.7%) knees exhibited mediopatellar plica (76 (22.2%) type A, 69 (20.2%) type B, and 18 (5.3%) type C) on MRI. Significant cross-sectional associations of MRI-detected mediopatellar plica and cartilage damage were observed for the medial patella (adjusted odds ratio (aOR) 2.12, 95% CI 1.23-3.64 for all types combined, and aOR 4.20, 95% CI 1.92-9.19 for type B lesion), but not for the anterior medial femur or the lateral PFJ. No associations were found between the presence of MRI-detected mediopatellar plica and BMLs in any patellofemoral subregion.

**Conclusion:**

On MRI, types A and B mediopatellar plicae were commonly observed in this cohort of subjects with knee pain. MRI-detected mediopatellar plica was cross-sectionally associated with higher likelihood of the presence of MRI-detected medial patellar cartilage damage after adjustment for confounders.

## Background

The mediopatellar plica, also known as the medial synovial shelf, plica synovialis mediopatellaris or plica alaris elongata, is a synovial fold representing an embryonic remnant from the developmental process of the synovial cavity formation in the knee [1]. It can be directly visualized by arthroscopy, but can also be evaluated non-invasively using conventional MRI. Sakakibara arthroscopically classified the mediopatellar plicae into four types (A - D) on the basis of size, which is currently the universally accepted classification scheme amongst orthopedic surgeons and radiologists [2-4].

Asymptomatic synovial plicae may be found within structurally normal knee joints. However, direct trauma, repetitive sports activities, or other pathologic knee conditions may provoke secondary inflammation in the synovial tissues surrounding the plica, and may result in increasing fibrotic changes, loss of elasticity, and various degrees of synovitis [4,5]. Owing to the anatomic location of the mediopatellar plica, a loss of normal elasticity can cause impingement of the plica between the medial femoral condyle and the medial facet of the patella during flexion-extension motion of knee [6]. A high frequency of degenerative chondral lesions on the facing medial patella and femoral condyle was observed in patients with mediopatellar plicae in arthroscopic studies [6,7].

Several studies have shown that anatomical variants/malalignment of the PFJ such as patellar alta, medial patellar inclination and trochlear dysplasia are associated with an increased risk of MRI-detected cartilage loss in the medial PFJ [8-11]. However, to the authors’ knowledge, there has been no MRI-based study that examined the frequency of the mediopatellar plica and its association with structural changes in the PFJ, although there have been several reports based on arthroscopic findings. Bone marrow changes, a finding commonly observed in conjunction with cartilage damage and osteoarthritis, can be studied with MRI but not with arthroscopy. Associations between MRI-detected cartilage damage and bone marrow lesions (BMLs) in the knee, including the PFJ, have been reported [12,13]. Thus, BMLs may also potentially be associated with the mediopatellar plica. Anatomical variants have been shown to be associated with MRI-assessed cartilage damage and BMLs [9,14]. It is unknown if the MRI-detected plica by itself has a relevant impact on the structural changes or if they are a result of the anatomical variants.

Aim of the present study was to examine the prevalence of mediopatellar plica on MRI in persons with chronic frequent knee pain and to investigate the cross-sectional association of knee pain with MRI-detected structural damage in the PFJ, i.e. cartilage damage and BMLs.

## Methods

### Study sample

Subjects included in the present study were participants in the Joints On Glucosamine (JOG) study (clinical trial registration number: NCT00377286). The JOG study is a 6-month, double-blind, randomized controlled trial to examine the efficacy of oral glucosamine supplementation. Two hundred and one participants, aged 35 to 65 with mild to moderate chronic, frequent knee pain (Western Ontario and McMaster Universities (WOMAC) score ≥ 125 and ≤500 [15]), were recruited at the University of Pittsburgh, PA, USA. Subjects were excluded from JOG if they screened positive for rheumatoid arthritis; had ankylosing spondylitis, psoriatic arthritis, chronic reactive arthritis; or renal insufficiency that required hemo- or peritoneal dialysis; were taking bisphosphonates or dietary supplements for knee pain in the 6 months prior to study entry; had a history of cancer (except for non-melanoma skin cancer); had or planned to have bilateral knee replacement surgery; or were unable to walk without assistance.

The baseline and follow-up MRI examinations of 346 knees of the 177 subjects who completed the study were examined. Although the JOG study itself was a longitudinal study, the present study involved only cross-sectional analyses of the baseline MRI examinations. Because of the image degradation due to motion artifacts four knees were excluded. As a result, 342 knees of the 177 subjects were included in the analyses.

Institutional Review Board approval at the University of Pittsburgh and written informed consent from all participants were obtained for the present study.

### Magnetic resonance imaging acquisition

MRI of each knee was performed using a 3 Tesla MR system (Siemens Trio, Erlangen, Germany). The protocol used for the Osteoarthritis Initiative was applied in the JOG study, excluding the fast low angle shot sequence and the multi-echo spin echo T2 mapping sequence. Details of the full Osteoarthritis Initiative pulse sequence protocol and the sequence parameters have been published [16]. The protocol included the sagittal triplanar three-dimensional dual echo at steady state (3D DESS) sequence (slice thickness = 0.7 mm, interslice gap = 0 mm, repetition time = 16.3 ms, echo time = 4.7 ms, flip angle = 25°, field of view = 140 mm × 140 mm, matrix = 384 × 307 pixels, echo train length = 1, number of slices = 35, bandwidth = 185 Hz/pixel, number of excitations = 1, anterior/posterior phase encoding axis, acquisition time = 10 minutes 23 seconds) and the sagittal intermediate-weighted fat-suppressed (IW FS) sequence (slice thickness = 3 mm, interslice gap = 0 mm, repetition time = 30 ms, echo time = 3,200 ms, flip angle = 180°, field of view = 160 mm × 160 mm, matrix = 313 × 448 pixels, echo train length = 5, number of slices = 37, bandwidth = 248 Hz/pixel, number of excitations = 1, anterior/posterior phase encoding axis, acquisition time = 4 minutes 42 seconds). Axial and coronal images were reformatted from the sagittal 3D DESS images.

### Magnetic resonance imaging assessment

All MRI assessments were performed blinded to clinical information of subjects using digital imaging software (eFilm Workstation, version 2.1.2, Merge Healthcare, Milwaukee, WI).

#### Mediopatellar plicae evaluation

**Figure 1:**
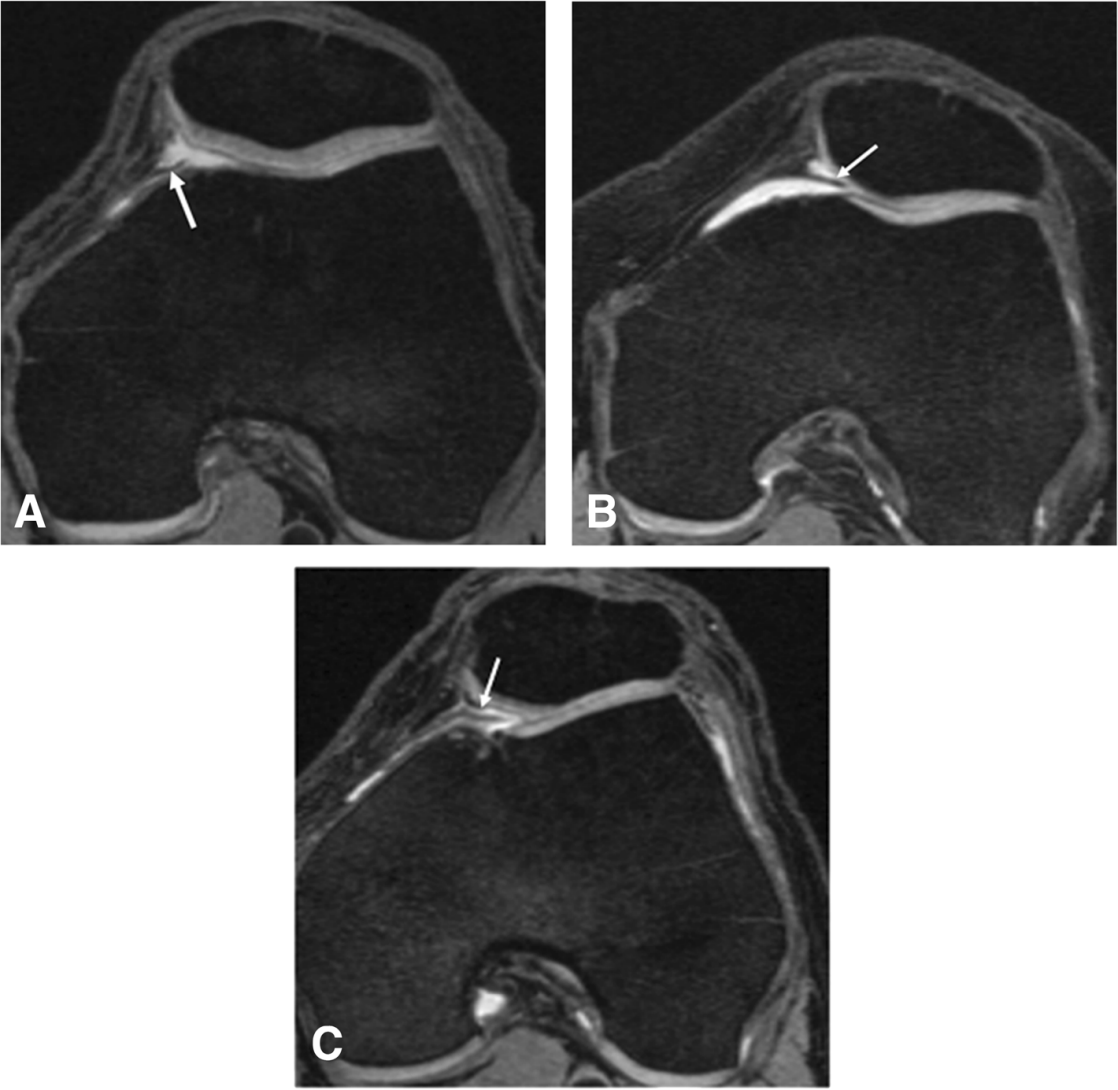
**MRI classification scheme of mediopatellar plicae modified from the Sakakibara arthroscopic classification. (A)** Type A lesion in the left knee consisting of a cord-like elevation in the synovial wall; **(B)** Type B lesion in the left knee, which has a shelf-like appearance but does not cover the anterior surface of the medial trochlea; **(C)** Type C lesion in the left knee, which has a large shelf-like appearance and covers the anterior surface of the medial trochlea.

From the baseline MRI of each knee, mediopatellar plica was scored by a musculoskeletal radiologist (LX) with 6 years of experience in musculoskeletal radiology and 1 year of research experience using semiquantitative scoring of knee MRI. Based on the Sakakibara arthroscopic classification [2], we developed an MRI grading system for the present study, which took into account the size of the mediopatellar plica in relation to the anterior medial trochlea. In our MRI grading system, mediopatellar plica was scored according to their size: 0 = no obvious mediopatellar plica; 1 = corresponds to Sakakibara Type A lesion consisting of a cord-like elevation in the synovial wall; 2 = corresponds to Type B lesion which has a shelf-like appearance but does not cover the anterior surface of the medial trochlea; 3 = corresponds to Type C lesion which has a large shelf-like appearance and covers the anterior surface of the medial trochlea (Figure 1). Although the Sakakibara classification also includes a Type D lesion (fenestrated plica), these type D lesions are rare and no comparative arthroscopic-MRI studies are available that have characterized these lesions on MRI [2,3].

#### Patellar alignment measurements

**Figure 2:**
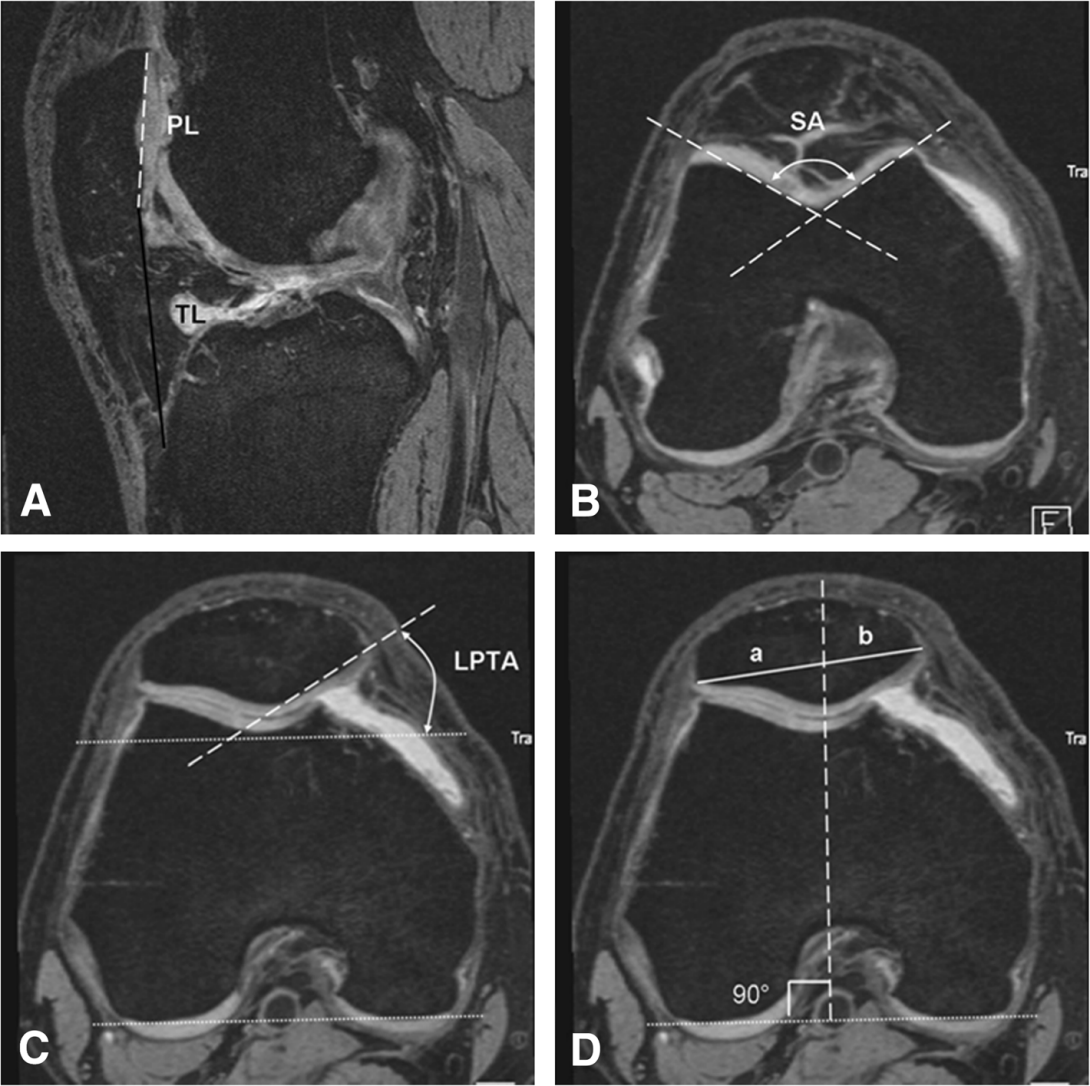
**Schema of measured patellar alignment indices. (A)** The sagittal slice referring to the middle of medial patellar facet: patellar length (PL, white broken line), patellar tendon length (TL, black line) and patellar length ratio (PLR = PL/TL) were measured; **(B)** On the axial slice at the level of superior 1/3 of trochlea, sulcus angle (SA) was measured; **(C)** and **(D)** On the specific axial slice that was defined by the bisecting line between the superior and inferior osseous patellar pole, lateral patellar tilt angle (LPTA) and bisect offset (BO = a*100/(a + b)) were measured.

Of the 342 knees, patellar alignment was not measurable in 9 knees due to patellar dysplasia, anterior cruciate ligament (ACL) tears or susceptibility artifacts resulting from previous ACL reconstruction surgeries. These 9 knees were excluded from the analysis. The alignment measurements of the PFJ were performed on the baseline MRI of 333 knees by one radiologist (LX) who also evaluated the mediopatellar plica according to the Insall and Salvati method [17]. On the sagittal slice at the middle of the medial patellar facet, patellar length (PL) and patellar tendon length (TL) were measured and the patellar length ratio (PLR = PL/TL) was then calculated (Figure 2A). On the axial slice through the level of the superior 1/3 of the trochlea, sulcus angle (SA) was measured as the angle between two lines, one connecting the lowest point of the trochlear sulcus and the medial trochlear bony margin, and the other connecting the lowest point of the trochlear sulcus and the lateral trochlear bony margin (Figure 2B). Lateral patellar tilt angle (LPTA) and bisect offset (BO) were measured on the specific axial slice defined by the bisecting line between the superior and inferior osseous patellar pole. LPTA is the angle between the posterior condylar line and the line drawn through the lateral inferior bony margin of the patella (Figure 2C). For BO measurement, two lines were drawn: one line connecting the medial and lateral poles of the patella and a vertical line perpendicular to the posterior condylar line and across the lowest point of the trochlear sulcus. The first line is bisected by the vertical line, the sections representing (a) the distance between the lateral border of the patella and the intersection point of the two lines and (b) the distance between the medial border of the patella and the intersection of the two lines (Figure 2D). BO was calculated according to the formula: BO = a*100/(a + b).

#### Semiquantitative assessment of structural patellofemoral joint damage

**Figure 3:**
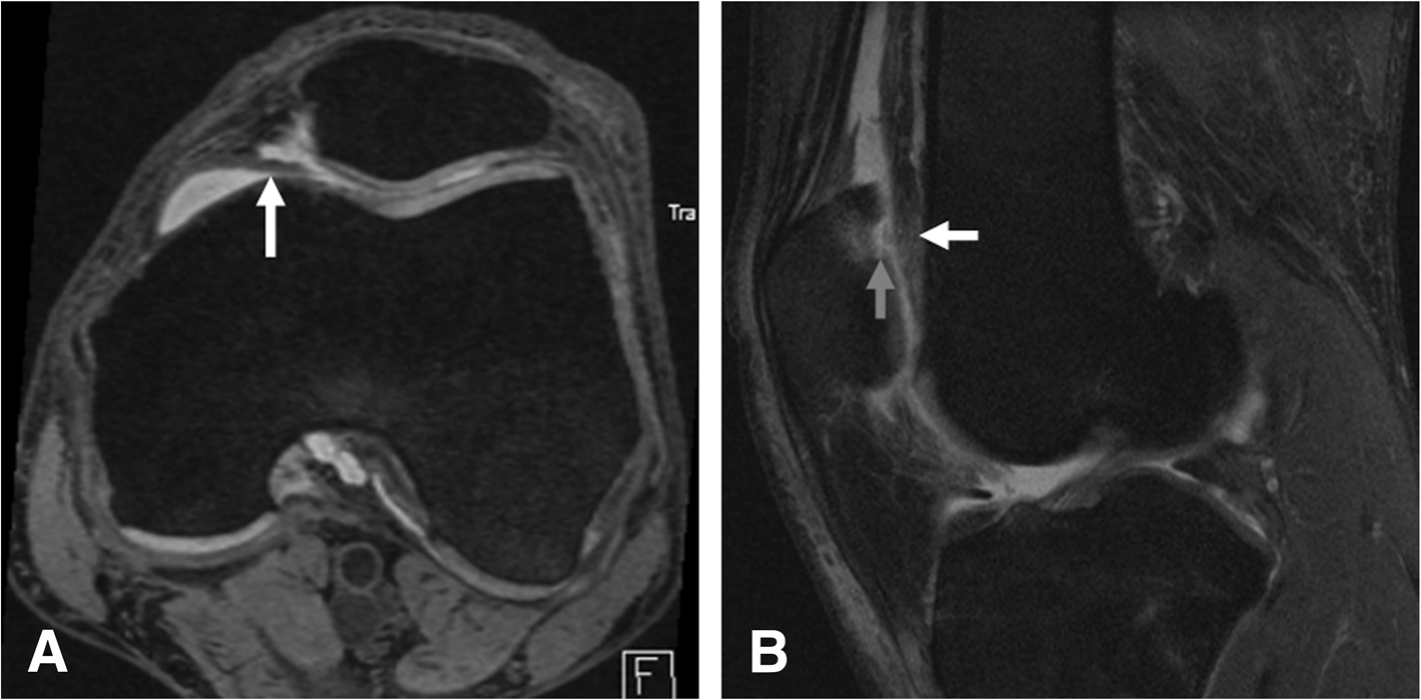
**Type C mediopatellar plica with cartilage damage and BMLs in the medial patella. (A)** Axial dual echo steady-state (DESS) image shows a type C mediopatellar plica (white arrow). **(B)** Sagittal intermediate-weighted fat-suppressed (IW FS) image shows a WORMS grade 2.5 full thickness focal defect (white arrow) and a corresponding WORMS grade 1 BML in the medial patella (grey arrow).

**Figure 4:**
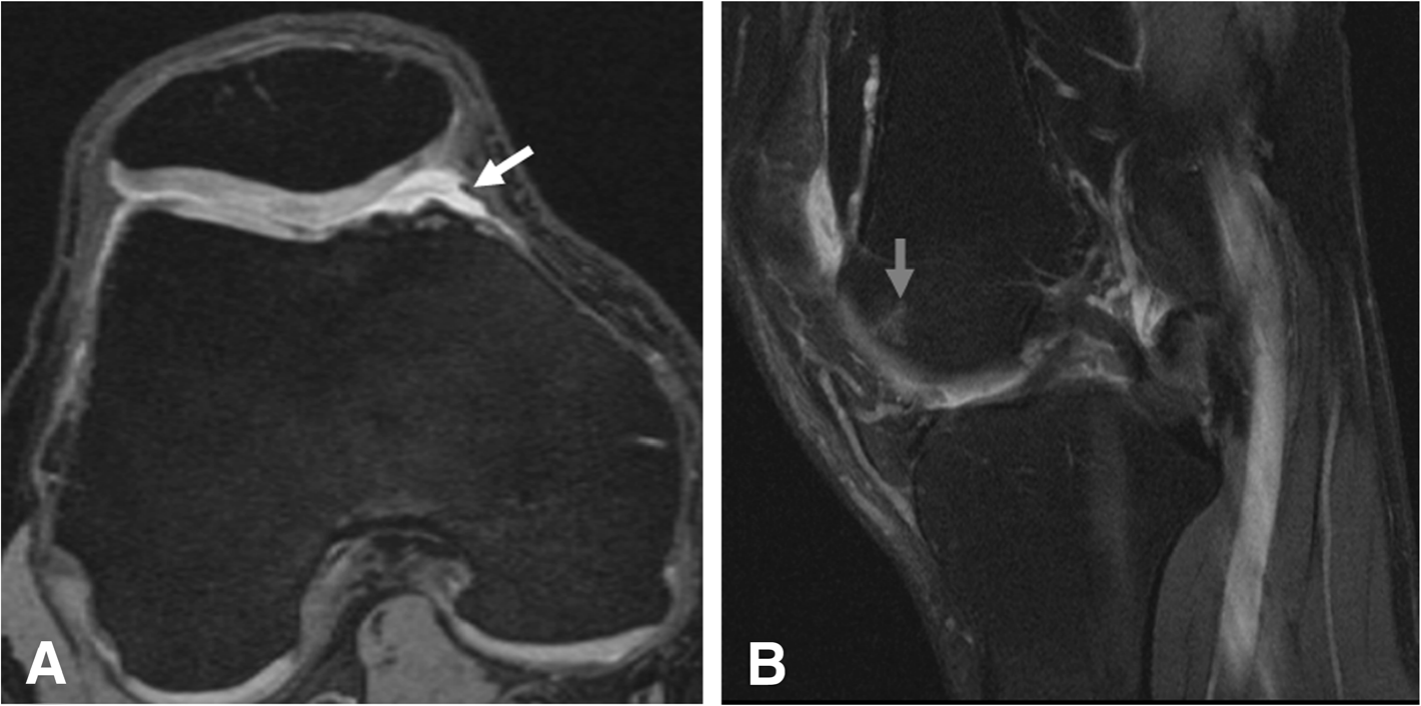
**Type A mediopatellar plica with a bone marrow lesion (BML) in the anterior medial femur. (A)** Axial dual echo steady-state (DESS) image shows a type A mediopatellar plica (white arrow). **(B)** Sagittal intermediate-weighted fat-suppressed (IW FS) image shows a WORMS grade 1 BML in the anterior medial femur (grey arrow head), a finding that was not associated with mediopatellar plica.

Severity of cartilage damage, subchondral BMLs, Hoffa-synovitis and effusion synovitis at baseline were semiquantitatively assessed for all knees (n = 342) by another musculoskeletal radiologist (FWR) with 7 years experience of semiquantitative assessment of knee MRI, using the Whole Organ Magnetic Resonance Imaging Score, (WORMS) [18]. Cartilage damage and BMLs were assessed in the articular subregions according to WORMS: the medial patella, the lateral patella, the anterior medial femur and the anterior lateral femur. Using the axial, sagittal, and coronal DESS images, and the sagittal IW FS images, cartilage damage was scored from 0 to 6 based on the thickness of cartilage loss and the extent of regional involvement (0 = normal thickness; 1 = focal swelling of cartilage without thickness loss; 2.0 = partial-thickness focal defect < 1 cm in greatest width; 2.5 = full-thickness focal defect <1 cm in greatest width; 3 = multiple areas of partial-thickness (grade 2.0) defects intermixed with areas of normal thickness, or a grade 2.0 defect wider than 1 cm but <75% of the region; 4 = diffuse (≥75% of the region) partial-thickness loss; 5 = multiple areas of full-thickness loss (grade 2.5) or a grade 2.5 lesion wider than 1 cm but <75% of the region; 6 = diffuse (≥75% of the region)) (Figure 3B). On the sagittal IW FS images, subchondral BMLs were scored from 0 to 3 based on the extent of subregional marrow involvement (0: none; 1: < 25% of the region; 2: 25-50% of the region; 3: >50% of the region). Effusion-synovitis was graded from 0 to 3 in regard to the estimated maximum distention of the synovial cavity (0: normal; 1: < 33% of maximum potential distention; 2: 33–66% of maximum potential distention; 3: > 66% of maximum potential distention) (Figure 4B). Hoffa-synovitis at the infrapatellar and intercondylar sites was graded 0 to 3 according to the degree of signal changes in Hoffa’s fat pad (0 = none; 1 = mild; 2 = moderate; 3 = severe).

### Statistical analysis

We collapsed WORMS grades of MRI assessment features (cartilage damage, BMLs, effusion synovitis and Hoffa-synovitis) into absent or present (WORMS grade ≥ 2 for cartilage damage and ≥ 1 for all other features). We considered a grade 1 cartilage lesion to be within normal limits, as these changes represent intrachondral signal changes of unknown clinical relevance with an intact articular surface. Logistic regression was performed to assess the cross-sectional relationships between the presence of mediopatellar plica and cartilage damage as well as BMLs in each compartment of 333 knees. Adjustment for confounders of PFJ structural damage was performed for the following: patellar alignment (PLR, LPTA, BO, and SA, as described above), age, gender, body mass index (BMI), and Hoffa- and effusion-synovitis [19]. For covariate adjustment, BMI was categorized as 1: < 25 kg/m^2^, 2: 25-30 kg/m^2^ and 3: > 30 kg/m^2^. We did not adjust for malalignment of the tibiofemoral knee joint (i.e. varus and valgus) because a recent large-scale study showed a lack of significant difference in the prevalence of medial PFJ cartilage damage in varus, valgus and neutrally aligned knees. All statistical analyses were performed using SAS software (Version 9.2 for Windows; SAS Institute, Cary, NC).

## Results

The present study included 177 participants with a mean age of 52 years (range: 35 to 65 years, standard deviation (SD) ± 6) and a mean BMI of 29 kg/m^2^ (SD ± 4). The percentages of overweight (25-30 kg/m^2^) and obese (> 30 kg/m^2^) subjects were 40.1% (71) and 41.2% (73), respectively. Subjects were predominantly white (90.4%, 160) and approximately half were women (46.3%, 82). Of the 342 knees, 163 (47.7%) knees exhibited mediopatellar plica. Of the 163 mediopatellar plicae, 46.6% (76/163) were type A, 42.3% (69/163) were type B, and 11.0% (18/163) were type C, according to our MRI grading scheme. The Kellgren and Lawrence grades for the 342 knees were: grade 0 = 105 knees (30.7%), grade 1 = 30 (8.8%), grade 2 = 38 (11.1%), grade 3 = 153 (44.7%), and grade 4 = 16 (4.7%).

In the medial patella, the frequency of any MRI-detected cartilage damage was 66.3% for knees with mediopatellar plica and 58.1% for knees without mediopatellar plica (Table 1). In the anterior medial femur, the frequency of any MRI-detected cartilage damage was 42.3% for knees with mediopatellar plica and 37.4% for knees without mediopatellar plica. The frequency of BMLs for knees with and without mediopatellar plica was 26.4% and 29.6% in the medial patella and 18.4% and 16.8% in the anterior medial femur, respectively (Table 2).

**Table 1:** Frequency of MRI-detected cartilage damage (WORMS grade ≥ 2) in the patellofemoral joint stratified by the presence of MRI-detected mediopatellar plica

**Compartment**	**Total**	**Without mediopatellar plica**	**With mediopatellar plica**			
			**All types**	**Type A**	**Type B**	**Type C**
	**(N = 342)**	**(N = 179)**	**N = 163**	**N = 76**	**N = 69**	**N = 18**
Medial patella	212 (62.0%)	104 (58.1%)	108 (66.3%)	44 (57.9%)	55 (79.7%)	9 (50.0%)
Lateral patella	141 (41.2%)	80 (44.7%)	61 (37.4%)	19 (25.0%)	30 (43.5%)	12 (66.7%)
Anterior medial femur	136 (39.8%)	67 (37.4%)	69 (42.3%)	28 (36.8%)	33 (47.8%)	8 (44.4%)
Anterior lateral femur	90 (26.3%)	49 (27.4%)	41 (25.2%)	15 (19.7%)	20 (29.0%)	6 (33.3%)

**Table 2:** Frequency of bone marrow lesions (BMLs) in the patellofemoral joint stratified by the presence of MRI-detected mediopatellar plica

**Compartment**	**Total**	**Without mediopatellar plica**	**With mediopatellar plica**			
			**All types**	**Type A**	**Type B**	**Type C**
	**(N = 342)**	**(N = 179)**	**N = 163**	**N = 76**	**N = 69**	**N = 18**
Medial patella	96 (28.1%)	53 (29.6%)	43 (26.4%)	13 (17.1%)	23 (33.3%)	7 (38.9%)
Lateral patella	101 (29.5%)	58 (32.4%)	43 (26.4%)	16 (21.1%)	20 (29.0%)	7 (38.9%)
Anterior medial femur	60 (17.5%)	30 (16.8%)	30 (18.4%)	14 (18.4%)	12 (17.4%)	4 (22.2%)
Anterior lateral femur	67 (19.6%)	34 (19.0%)	33 (20.3%)	10 (13.2%)	17 (24.6%)	6 (33.3%)

There was a strong cross-sectional association between the presence of MRI-detected mediopatellar plica (all types combined and type B) and medial patellar cartilage damage, with an adjusted odds ratio (aOR) of 2.12 (95% confidence interval (CI), 1.23-3.64) (Table 3). No significant association between the presence of MRI-detected mediopatellar plica and cartilage damage was detected for the anterior medial femur or the lateral PFJ subregions. There was no significant cross-sectional association between the presence of MRI-detected mediopatellar plica and BMLs in any subregion of the PFJ (Table 4).

**Table 3:** Cross-sectional association of MRI-detected mediopatellar plica with cartilage damage (WORMS grade ≥ 2) in the patellofemoral joint

**Compartment**	**Total**	**Without mediopatellar plica**	**With mediopatellar plica**			
			Adjusted odds ratio (95% confidence interval)**			
			**All types**	**Type A**	**Type B**	**Type C**
	**(N = 333)**	**(N = 179)**	**N = 159**	**N = 76**	**N = 69**	**N = 18**
Medial patella	96	1.00	2.12*	1.87	4.20*	0.68
	(28.1%)	(Reference)	**(1.23–3.64)**	(0.94-3.71)	**(1.92-9.19)**	(0.23-2.06)
Lateral patella	101	1.00	1.06	0.65	1.45	2.36
	(29.5%)	(Reference)	(0.62-1.81)	(0.31-1.38)	(0.71-2.99)	(0.70-8.03)
Anterior medial femur	60	1.00	1.17	1.02	1.21	1.86
	(17.5%)	(Reference)	(0.70-1.96)	(0.52-2.00)	(0.62-2.39)	(0.58-5.94)
Anterior lateral femur	67	1.00	1.19	1.07	1.21	1.31
	(19.6%)	(Reference)	(0.65-2.20)	(0.48-2.40)	(0.54-2.71)	(0.38-4.54)

*Numbers in boldface represent statistically significant results with p<0.05.

**Adjustment for age, gender, BMI, PLR, SA, LPTA, BO, effusion synovitis, Hoffa’s synovitis.

NB) 9 knees without measurement of malalignment were excluded.

**Table 4:** Cross-sectional association of MRI-detected mediopatellar plica with bone marrow lesions (WORMS grade ≥ 1) in the patellofemoral joint

**Compartment**	**Total**	**Without mediopatellar plica**	**With mediopatellar plica**			
			Adjusted odds ratio (95% confidence interval)*			
			**All types**	**Type A**	**Type B**	**Type C**
	**(N = 333)**	**(N = 179)**	**N = 159**	**N = 76**	**N = 69**	**N = 18**
Medial patella	96	1.00	1.35	0.79	1.90	1.89
	(28.1%)	(Reference)	(0.77 – 2.34)	(0.36-1.71)	(0.94-3.80)	(0.63-5.67)
Lateral patella	101	1.00	1.17	1.00	1.20	1.59
	(29.5%)	(Reference)	(0.67 – 2.04)	(0.47-2.15)	(0.58-2.50)	(0.51-4.95)
Anterior medial femur	60	1.00	0.95	0.92	0.66	2.75
	(17.5%)	(Reference)	(0.51 – 1.78)	(0.41-2.04)	(0.28-1.54)	(0.71-10.68)
Anterior lateral femur	67	1.00	1.55	1.02	1.62	2.97
	(19.6%)	(Reference)	(0.81 – 2.99)	(0.41-2.51)	(0.71-3.68)	(0.86-10.23)

*Adjustment for age, gender, BMI, PLR, SA, LPTA, BO, effusion synovitis, Hoffa’s synovitis.

NB) 9 knees without measurement of malalignment were excluded.

## Discussion

In summary, type A and B mediopatellar plicae were much more commonly observed on MRI than type C plicae. There was a strong cross-sectional association between the presence of MRI-detected mediopatellar plica and medial patellar cartilage damage but not in other subregions of the PFJ. We did not find any significant cross-sectional association between the presence of MRI-detected mediopatellar plica and BMLs in any subregion of the PFJ.

The prevalence of mediopatellar plica has been reported with wide variation, ranging from 18.5 to 80%. Most data were collected from knee arthroscopic surgery on patients with knee symptoms or trauma [3,6,7]. According to a recent large-scale retrospective study performed by Nakayama and colleagues, 79.9% of 3889 symptomatic knees were found to have mediopatellar plica confirmed by arthroscopic surgery [3]. In their study, the incidence of Sakakibara type A, B, C and D mediopatellar plicae was 35.2%, 22.4%, 12.3% and 10.0%, respectively. We found similar numbers for the frequency distribution of the different types of MRI-detected mediopatellar plicae following the same trend (A > B > C) in our cohort. Another large-scale retrospective study by Lyu and colleagues based on arthroscopic evaluation found 472 (29.7%) of 1587 symptomatic knees had mediopatellar plica [6]. Christoforakis and colleagues prospectively evaluated 1000 symptomatic knees using arthroscopy and found 321 (32.1%) knees exhibiting a mediopatellar plica [7]. Different definitions of mediopatellar plica exist: some authors describe a small fold of synovium as a plica and others describe it as absent, and reported prevalence has to be interpreted carefully [1]. Further, variation in study populations contributes to the wide range of prevalence estimates reported in the literature, and the present study population included mainly overweight and obese adults with knee pain in whom tibiofemoral OA was also highly prevalent.

Our findings are based solely on MRI findings, and there can potentially be a discrepancy between our findings and arthroscopic findings with respect to evaluation of mediopatellar plica. Thus, results of our study need to be interpreted with caution when comparing them to arthroscopy-based studies. Studies reporting the prevalence of mediopatellar plica on MRI are limited, but MRI seems to be sensitive for the detection of mediopatellar plica [4,20]. According to a report by Jee and colleagues, the sensitivity of MRI for detection of mediopatellar plica, using arthroscopic findings as the gold standard, was 71-95%, and the specificity 72-83%, depending on the pulse sequence used [21]. A study by Nakanishi and colleagues showed 27 (93.1%) of the 29 arthroscopy-detected mediopatellar plica were also detected by MRI [20]. Boles and colleagues reported that 46 (69.7%) of 66 symptomatic knees demonstrated a mediopatellar plica on MRI [22]. Consistent with the present study, then, the presence of mediopatellar plica, whether it be detected by MRI or arthroscopy, seems to be fairly common in symptomatic knees.

Mediopatellar plica may cause snapping and impingement within the medial PFJ during knee motion and are believed to contribute to degenerative chondral lesions in the medial PFJ compartment [5,6,23,24]. Lyu and colleagues found that 97% of the symptomatic knees with arthroscopy-detected mediopatellar plica had degenerative cartilage damage on the edge and the anterior part of the medial femoral condyle [6]. Furthermore, the severity of the degenerative cartilaginous lesions was positively correlated with the severity of the pathologic changes of the mediopatellar plica [6]. Christoforakis and colleagues demonstrated a significantly increased incidence of cartilage damage in the medial patella (47.7% vs 27.5%, *P* < 0.001) and the anterior medial femoral condyle (80.2% vs 45.0%, *P* < 0.001) in patients with arthroscopy-detected mediopatellar plica compared to those without, and an association of larger and more fibrotic plica with larger cartilage lesions was observed [7].

In contrast, in our study the presence of any type of MRI-detected mediopatellar plica correlated significantly with cartilage damage at the medial patellar facet (aOR: 2.11, 95% CI: 1.23-3.62) but not in the anterior medial femur (aOR: 1.23, 95% CI: 0.80-1.89). Compared to reports by Christoforakis and colleagues [7], we found a higher frequency of cartilage damage in the medial patella for knees with and without mediopatellar plica, and a much lower frequency of cartilage damage in the anterior medial femur for knees with mediopatellar plica. However, Christoforakis’s subjects were symptomatic younger patients (mean age, 37.4 years) who underwent arthroscopy because of knee pain, locking or instability, and one third of them had a history of knee injury [7]. Thus, their study sample is not comparable to ours and may explain the discrepancy in the frequency of cartilage damage in the medial trochlea compared to our study. Other studies reported associations between mediopatellar plica and cartilage lesions in the anterior medial femur [6,7,25]. The lack of this finding in our study may be attributed to the presence of a relatively small number of large mediopatellar plica in our cohort, and possibly to the fact that our study is solely based on MRI which has a different diagnostic performance for detection of plica compared to arthroscopy.

The size and morphology of mediopatellar plica seem to have a role in femoral impingement and chondral damage [22,26]. The cross-sectional association between the mediopatellar plica and medial patellar cartilage damage detected in the present study suggests that medial patellar cartilage is more susceptible to the mechanical abrasion caused by the mediopatellar plica than the anterior medial femur. Localized synovitis might play an additional role, but we did not assess synovitis at separate locations other than those described. Further, without contrast-enhanced MRI, synovitis cannot be visualized adequately [27,28]. In line with the literature, we found that coexistence of the mediopatellar plica and cartilage damage in the medial patella seems to be a common finding in symptomatic knees. Thus, close evaluation of the medial patellar cartilage and evaluation for the presence of a mediopatellar plica should be part of routine MRI assessment of symptomatic knees.

In a cross-sectional analysis with MRI-detected cartilage damage, type A plicae showed a borderline significant increase in the adjusted odds ratio (aOR) while type B lesions showed a significant increase in the aOR. From these results, it remains unclear whether patients with a type A (i.e. very small) mediopatellar plica might benefit from 'prophylactic’ treatment or could be safely left alone. A clinically relevant question to ask here is whether the presence of type A (and also B and C) plica would cause future cartilage loss or progression of pre-existing cartilage damage. Although we would have liked to do longitudinal analyses, we did not have sufficient numbers of each type of plicae that showed longitudinal changes over the 6-month follow-up period of the parent JOG study.

To the authors’ knowledge, this is the first study to examine the association between MRI-detected mediopatellar plica and BMLs in the PFJ. BMLs in OA represent subchondral bone changes as a result of localized increased loading [29]. Pathologically, mediopatellar plica behaves like bowstrings leading to impingement or abrasion over the medial PFJ compartment [26]. We found an association bewteen the presence of MRI-detected mediopatellar plica and cartilage damage in the medial patellar facet but such association was not observed for subchondral BMLs. Since cartilage damage is commonly associated with BMLs, one could also expect a higher prevalence of BMLs at the medial patella. There is little data on BMLs in the PFJ but recent longitudinal data from the JOG study showed that risk factors for short term progressive cartilage damage differ between the TFJ and the PFJ [19]. In the study, BMLs strongly predicted cartilage loss in the TFJ but not the PFJ, suggesting a different role of BMLs in regard to structural progression and associated localized joint damage. Our findings imply that BMLs are probably a consequence of compressive overloading (which applies to tibiofemoral joint) rather than frictional wear (which applies to patellofemoral joint). Lateral trochlear inclination and quadriceps weakness have been associated with cartilage damage and BMLs in the lateral PFJ compartment [10,11]. In the medial PFJ compartment, quadriceps weakness was found to increase risk only of cartilage damage [10]. We did not observe any statistically significant differences in aORs for the presence of BMLs with any type of mediopatellar plicae (Table 4). Longitudinal analysis of BMLs was not possible because too few knees exhibited longitudinal changes.

There are several limitations of the present study. A major limitation is that only a cross-sectional relationship between the presence of mediopatellar plica and cartilage damage and BMLs was examined. Although the JOG study is a 6-month longitudinal study, only a very few cases (less than 5%) were detected with progression of cartilage damage or BMLs in the PFJ at the follow-up visit [19]. Another limitation is that the cohort of this study is heterogeneous and relatively small, and moreover all subjects had chronic knee pain but not necessarily knee OA. One cannot discuss the actual prevalence of mediopatellar plica in the population based on this selected cohort of subjects with knee pain. A much lower frequency of type C plica compared to types A and B is also a limitation, as it is likely that the number of type C lesions did not give us sufficient statistical power to make a meaningful assessment of aOR values. Since type C lesions are larger than type B lesions, an aOR value higher than those for types A or B lesions was expected but this was not the case in our study. Our hypothesis needs to be confirmed by a study with a much larger frequency of type C plica.

## Conclusions

In conclusion, in this cohort of subjects with knee pain, MRI-detected type A and B mediopatellar plicae were common, while type C plicae were rare. The presence of any type of mediopatellar plica on MRI was cross-sectionally associated with higher likelihood of the presence of MRI-detected medial patellar cartilage damage after adjustment for multiple structural and demographic risk factors of PFJ cartilage damage. A longitudinal study is warranted to assess whether the presence of baseline mediopatellar plica predicts worsening of cartilage damage and associated BMLs in the PFJ.

## Abbreviations

JOG: Joints on glucosamine; BML: Bone marrow lesion; MRI: Magnetic resonance imaging; TFJ: Tibiofemoral joint; PFJ: Patellofemoral joint; WOMAC: Western Ontario and McMaster Universities; DESS: Dual Echo at Steady State; IW FS: Intermediate-weighted fat-suppressed; WORMS: Whole organ magnetic resonance imaging score; OR: Odds ratio; aOR: Adjusted odds ratio; ACL: Anterior cruciate ligament; PL: Patellar length; TL: Patellar tendon length; PLR: Patellar length ratio; SA: Sulcus angle; LPTA: Lateral patellar tilt angle; BO: Bisect offset

## Competing interests

No authors have conflict of interest that may inappropriately bias this study within the last 3 years. However, we would like to disclose the following: AG is the President of Boston Imaging Core Lab (BICL) LLC, and a consultant to Merck Serono, Stryker, Genzyme, AstraZeneca, and Novartis. FWR is the CMO of BICL, LLC, and a consultant to Merck Serono and National Institute of Health. CKK received funding from AstraZeneca and the Beverage Institute, and is a consultant to Novartis.

## Authors’ contributions

All authors contributed to the study concepts and design. DH, LX, AG, MJH and FWR contributed to literature search. All authors contributed to the execution of this study, including participant recruitment and data acquisition and interpretation and analysis of images. CKK and MJH contributed to the statistical analysis. All authors contributed to manuscript preparation and editing and gave final approval for publication of this article. FWR is the guarantor of the integrity of the entire study.

## Pre-publication history

The pre-publication history for this paper can be accessed here:

http://www.biomedcentral.com/1471-2474/14/292/prepub
